# Protecting Healthcare Workers Amid the COVID-19 Crisis: A Safety Protocol in Wuhan

**DOI:** 10.3389/fpubh.2020.577499

**Published:** 2020-10-08

**Authors:** Yunlu Liu, Shijun Yang, Man Hung, Wei Tong, Yong Liu

**Affiliations:** ^1^Department of Orthopaedics, Union Hospital, Tongji Medical College, Huazhong University of Science and Technology, Wuhan, China; ^2^Department of Cardiology, Union Hospital, Tongji Medical College, Huazhong University of Science and Technology, Wuhan, China; ^3^College of Dental Medicine, Roseman University of Health Sciences, South Jordan, UT, United States

**Keywords:** COVID-19, SARS-CoV-2, Wuhan, healthcare workers, protocol

## Abstract

Coronavirus disease 2019 (COVID-19), which is caused by a distinct coronavirus, is an acute infectious disease that spreads mainly via the respiratory route. During the COVID-19 outbreak, many healthcare systems faced a severe burden when a large number of healthcare workers (HCWs) became infected due to the lack of adequate protection. Consequently, it was apparent that it is important to ensure the health and safety of HCWs in order to control the outbreak throughout society. In this article, we share our successful protocol for protecting the safety of HCWs in the course of their daily work in an orthopedics department with the aim of eventually reducing the risk of nosocomial infection. None of our HCWs or their families contracted the infection during the COVID-19 pandemic.

## Introduction

In December 2019, an outbreak of coronavirus disease 2019 (COVID-19), caused by severe acute respiratory syndrome coronavirus 2 (SARS-CoV-2), was first reported in Wuhan, China, and subsequently spread rapidly around the world (1). In order to control the COVID-19 spread, the Chinese government imposed a lockdown of Wuhan City on January 23, 2020 (2). The most common symptoms of SARS-COV-2 infection are fever, cough, shortness of breath, and myalgia or fatigue. SARS-CoV-2 is highly contagious and can be transmitted by droplets, via direct contact and possibly by aerosols (3). On March 11, 2020, the World Health Organization declared the disease a pandemic. As of August 5, 2020, there were 18,318,928 confirmed cases and 695,043 confirmed deaths reported globally (4).

During the early stage of the COVID-19 outbreak, many healthcare workers (HCWs) became infected due to lack of appropriate infection prevention and control protocols and implementation procedures. In Wuhan Union Hospital, 14 HCWs were infected by two index patients in the early days (5). In Italy, more than 3,300 HCWs had been infected, and at least 22 had died by early March, and by the end of March, COVID-19 had infected 20% of the frontline HCWs, and many had died (6). In the United States, by April 4, 2020 a total of 9,282 HCWs had been infected by COVID-19, including 27 deaths (7). However, in our hospital the infection became well-controlled once a strict safety protocol was implemented. Starting from January 25, 2020, our hospital was one of the designated hospitals for intensive care treatment of COVID-19 patients, yet none of the HCWs there was infected during the frontline medical care of COVID-19 patients following the implementation of the strict safety protocol.

At present, multiple countries are experiencing the COVID-19 pandemic, and HCWs are the most valuable resource in every country for saving patients' lives. Thus, it is imperative to protect the health and safety of HCWs during the COVID-19 pandemic so that they can treat patients. Fortunately, effective infection control measures have been put in place to protect the safety of HCWs in some countries (8–10). The aim of this article is to share our successful protocol, which ensured the safety of all of the HCWs in our orthopedics department and strengthened the overall COVID-19 epidemic control. This protocol covered five aspects: (1) safety protection classification, (2) reasonable working hours, (3) ward protection, (4) operating room protection, and (5) rest area protection. These aspects are presented individually in the following sections.

## Safety Protection Classification

Beginning on January 20, 2020, infection control training was provided to all medical staff at our hospital. The infection control measures implemented in our hospital's orthopedic department were based on detailed risk assessments by both local orthopedic and infection control experts. These measures were classified into a four-level hierarchy of control and were used in different risk environments for recommendation of personal protective equipment (PPE) usage for HCWs (Table 1).

**Table 1:** Infection control measures across protection levels.

**Protection level**	**Personal protective equipment**	**Recommended usage**
Level 0	White coats, surgical mask or N95 respirator, surgical cap	Clean area (office of healthcare worker)
Level I	Protective suits, surgical mask or N95 respirator, protective goggles, gloves, shoe covers, surgical cap	Ward rounds
Level II	Protective coveralls, N95 respirator, protective goggles, gloves (double), long shoe covers, surgical cap	Transferring patients, dressing change, stitches removed
Level III	Protective coveralls, N95 respirator and surgical mask, protective goggles, gloves (triple), long shoe covers, surgical cap, powered air purifying respirators	Contact with patients' blood, body fluids, and involvement in any aerosol-generating procedures

## Reasonable Working Hours

HCWs could have been infected due to the shortage of staff and lack of supplies resulting from the large influx of COVID-19 patients in the early stages (11, 12). Previous studies have demonstrated that severe fatigue can contribute to a higher probability of contracting COVID-19 in HCWs; thus, reducing workload could be a strategy for orthopedic surgeons to defend against becoming infected with COVID-19 (12). In our hospital, frontline medical staff were limited to work for 3 h in the quarantine ward and 8 h in the clean office area during a regular day. Ensuring that frontline medical staff have adequate rest time was a priority in the orthopedic department of our hospital.

## Ward Protection

**Figure 1:**
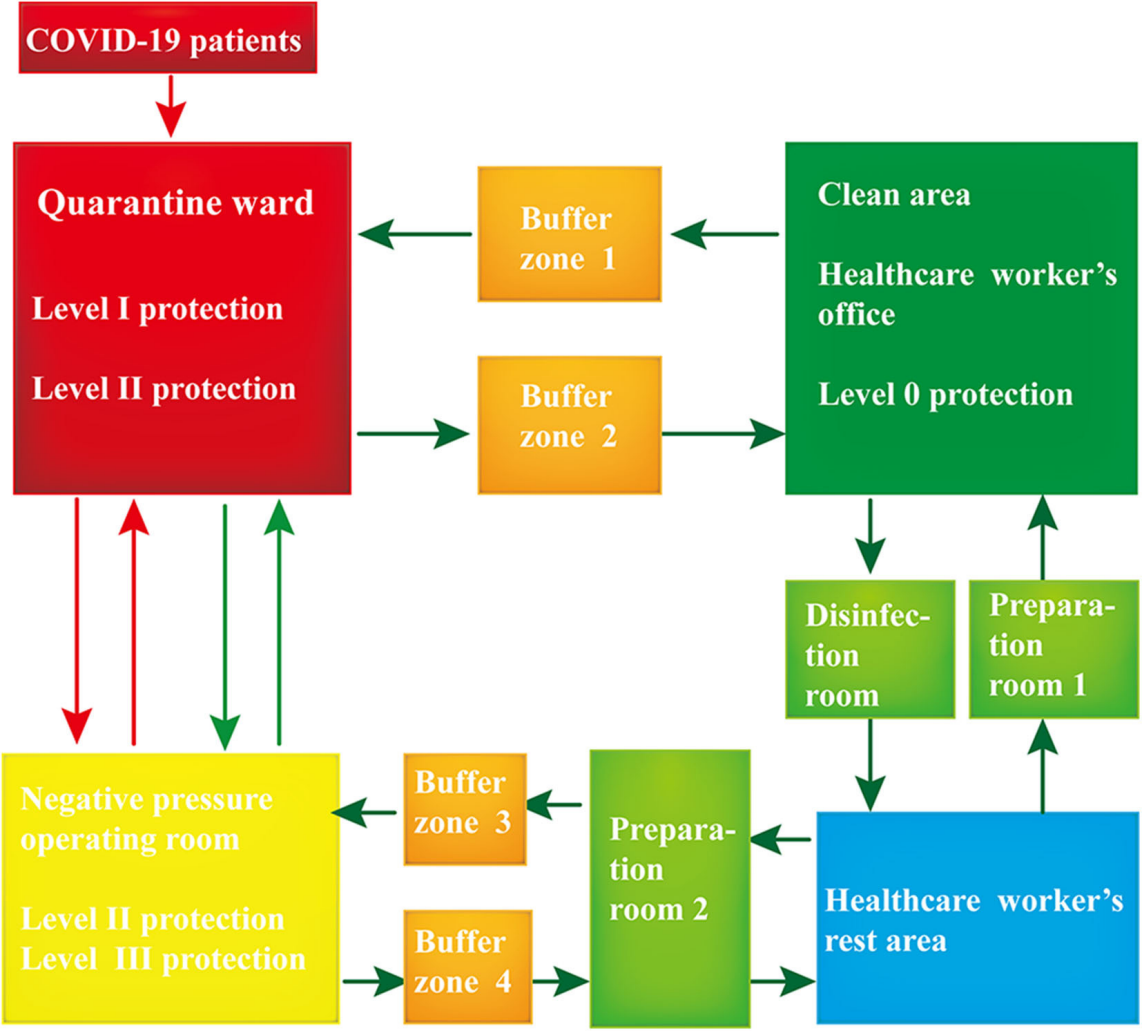
Conceptual scheme of workplace protection for healthcare workers (HCWs). The green arrows show the direction of movement of the HCWs; the red arrows show the direction of movement of the coronavirus disease 2019 (COVID-19) patients. The entrances to all rooms were marked with specific signs warning the HCWs to move in the right direction. Patients testing positive for COVID-19 were directed to the quarantine ward where they were placed in individual isolation rooms for further treatment. The clean area comprised the office where the medical staff worked, and measures were taken to prevent the virus from entering this area. The disinfection room was the place where the HCWs disinfected their personal belongings (cell phone, watch, and so on) and performed hand hygiene. The preparation room was the place where the HCWs dressed in appropriate personal protective equipment (PPE). The negative pressure operating room was designated for the surgical treatment of COVID-19 patients, and it is an effective measure to control the source of infection and block the route of transmission.

**Figure 2:**
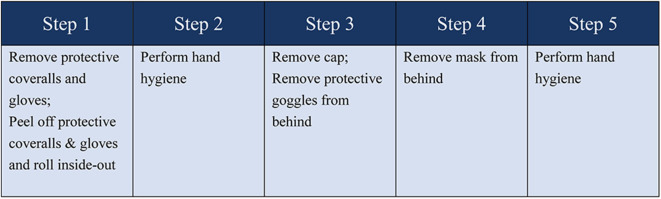
The order of removing personal protective equipment.

Our ward was divided into three areas, comprising the quarantine ward, buffer zone, and clean area (Figure 1). The COVID-19 patients' access to the ward was separate from that of the HCWs. The HCWs were obliged to wear appropriate PPE before entering the buffer zone. This procedure involved two HCWs working together to check for any damage to their PPE. After the HCWs entered buffer zone 1, they again helped each other to ensure that their PPE was properly secured in place. In the quarantine ward, the hands of the HCWs were disinfected with 75% alcohol before and after patient contact. Once their work in the quarantine ward was finished, the HCWs entered buffer zone 2 to remove their PPE. It was important that the PPE be removed following a given order (see Figure 2 for details). The HCWs then disinfected the contaminated PPE in the buffer room. Subsequently, the HCWs put on a new surgical mask, left the buffer zone, and entered the clean office area. It was important to ensure that the door into the buffer zone and the door out of the buffer zone could not be opened at the same time.

HCWs working in the clean area (i.e., level 0) only needed to wear white coats and N95 respirators (or surgical masks). We used throat swabs to sample items in the clean area regularly and removed any items having positive SARS-CoV-2 nucleic acid test results. We also ensured that the HCWs refrained from touching their eyes, nose, or mouth before performing hand hygiene. In addition, we required the HCWs to put their cell phones in a clear plastic protective bag to minimize accidental contamination of their cell phones.

It was essential to follow the principles of social distancing and avoid face-to-face contact with colleagues during meal times to minimize potential disease transmission. In our hospital, no more than three HCWs were allowed to eat at the same time in the dining area, and everyone must keep a distance of more than 1 m.

When the HCWs left the office area, they entered the disinfection room to change into a new mask, disinfect their personal belongings, and perform hand hygiene.

## Operating Room Protection

During the outbreak of COVID-19, elective surgeries had been suspended in many areas (13, 14). However, some patients affected by fracture and soft tissue injury required immediate assessment and emergency surgeries. In the department of orthopedics, we treated seven COVID-19 patients with fracture and one with lower limb ischemia and necrosis, of whom four patients underwent surgical treatment. None of the HCWs was infected as a result of caring for these COVID-19 patients.

The COVID-19 patients were transferred to a negative pressure operating room through a separate path and elevator by operative staff wearing PPE (level II). During the transfer, the patients wore a surgical mask. The surgeon entered preparation room 2, changed into an operating gown, performed surgical hand preparation, and dressed in the appropriate PPE (level III). After adequate safety examination and proper protection were secured in buffer zone 3, the surgeon entered the negative pressure operating room. The COVID-19 patient was anesthetized by an anesthesiologist wearing PPE (level II) in the negative pressure room. However, if the patient was under general anesthesia, which involved an open airway, the anesthesiologist must wear a full-face mask. The surgeon wore a full-face mask, disinfected his/her hands with 75% alcohol solution, and put on the first pair of sterile surgical gloves and sleeve protectors, followed by a sterile, disposable, surgical gown, and then a second pair of gloves. Subsequently, the surgeon placed the COVID-19 patient in an appropriate surgical position.

SARS-CoV-2 can be transmitted by droplets, direct contact, and possibly by aerosols. In orthopedic surgical procedures, the use of powered instruments, such as electrocautery, bone saws, reamers, and drills, releases aerosols (15). Therefore, in these high-risk procedures, it was necessary for us to minimize the number of surgical staff involved and to shorten the operation time as much as possible. Each operation room was equipped with skilled staff according to the operation type. The surgical staff were not allowed to leave the operating room, and the external staff could not enter the operating room until the operation was completed. All protective apparel and respirators were immediately discarded before leaving the operating room.

At the end of the surgical procedure, the surgeon would remove the outermost pair of gloves, the surgical gown, sleeve protectors, and full-face mask. His/her hands were disinfected with an alcohol solution, and then the surgical mask and the surgical cap were removed. Finally, the surgeon removed all his/her gloves and disinfected his/her hands before leaving the operating room. The PPE was removed in buffer zone 4, and hand hygiene was performed before entering preparation room 2 where the surgeon took a shower.

## Rest Area Protection

The rest area for HCWs was a hotel next to the hospital, which was requisitioned by the hospital. The hospital also guaranteed the availability of adequate food and daily living supplies for everyone in the hotel. HCWs in the hotel could take shuttle buses to and from the hospital. In addition, if an HCW was accidentally exposed to COVID-19, they would be required to leave the frontline and remain under quarantine for 14 days in the hotel. All the staff were advised to measure their own body temperatures daily and promptly report any symptoms of upper respiratory tract infection, vomiting, or diarrhea. Medical staff would also be quarantined in the hotel for 14 days when they left the frontline before returning home. In addition, a physical examination including pulmonary computed tomography, COVID-19 nucleic acid, and antibody testing are also needed. This ensured that the colleagues and families of HCWs could also be properly protected.

## Conclusions

With the rapid spread of COVID-19, many healthcare systems faced severe burdens. In the early stage, a large number of medical staff were infected due to the lack of adequate protection. Currently, the COVID-19 pandemic is evolving into more of a marathon and less of a short-lived sprint (16). Some experts have warned of a possible second wave of COVID-19 (17). In the long run, proper protection from contracting COVID-19 in clinics and hospitals is necessary and will likely become the norm. The protection of HCWs and appropriate training are of paramount importance in the fight against COVID-19. We hope our protocol of measures, which successfully controlled COVID-19 infection in our orthopedics department, can help HCWs minimize the risks of infection in medical facilities around the world.

## Author Contributions

YuL collected the data and drafted the manuscript. SY helped conceive the study and drafted the manuscript. MH contributed to language control and revised the manuscript. WT and YoL conceived the study, coordinated the study tasks, and helped draft the manuscript. All authors contributed to improvement of the manuscript and approved the submitted version.

## Conflict of Interest

The authors declare that the research was conducted in the absence of any commercial or financial relationships that could be construed as a potential conflict of interest.
