# Corrigendum: Protecting Healthcare Workers Amid the COVID-19 Crisis: A Safety Protocol in Wuhan

**DOI:** 10.3389/fpubh.2021.660323

**Published:** 2021-03-08

**Authors:** Yunlu Liu, Shijun Yang, Man Hung, Wei Tong, Yong Liu

**Affiliations:** ^1^Department of Orthopaedics, Union Hospital, Tongji Medical College, Huazhong University of Science and Technology, Wuhan, China; ^2^Department of Cardiology, Union Hospital, Tongji Medical College, Huazhong University of Science and Technology, Wuhan, China; ^3^College of Dental Medicine, Roseman University of Health Sciences, South Jordan, UT, United States

**Keywords:** COVID-19, SARS-CoV-2, Wuhan, healthcare workers, protocol

In the published article, there were errors in affiliations 1, 2. Instead of “^1^Department of Orthopaedics, Tongji Medical College, Union Hospital, Huazhong University of Science and Technology, Wuhan, China, ^2^Department of Cardiology, Tongji Medical College, Union Hospital, Huazhong University of Science and Technology, Wuhan, China,”, it should be “^1^Department of Orthopaedics, Union Hospital, Tongji Medical College, Huazhong University of Science and Technology, Wuhan, China, ^2^Department of Cardiology, Union Hospital, Tongji Medical College, Huazhong University of Science and Technology, Wuhan, China.”.

The authors apologize for this error and state that this does not change the scientific conclusions of the article in any way. The original article has been updated.

